# Real-world treatment patterns for patients 80 years and older with early lung cancer: a nationwide claims study

**DOI:** 10.1186/s12890-018-0699-0

**Published:** 2018-08-03

**Authors:** Kyungjong Lee, Hye Ok Kim, Hee Kyoung Choi, Gi Hyeon Seo

**Affiliations:** 1 Department of Medicine, Division of Pulmonary and Critical Care Medicine, Samsung Medical Center, Sungkyunkwan University School of Medicine, Seoul, South Korea; 2 Health Insurance Review and Assessment Service, 60 Hyeoksin-ro (Bangok-dong), Wonju-si, Gangwon-do 26465 South Korea

**Keywords:** Radiosurgery, Thoracic surgery, Treatment trends, Lung neoplasms

## Abstract

**Background:**

Old age is an important factor that could affect the treatment of early-stage lung cancer. In this study, we evaluated the treatment patterns and outcomes of patients over the age of 80 years who had been diagnosed with early-stage lung cancer in real-world practice.

**Methods:**

Elderly patients who were diagnosed with early-stage lung cancer between 2008 and 2016 were identified using claims data provided by the Health Insurance Review and Assessment Service. The proportion of patients who underwent surgical resection or stereotactic body radiation therapy (SBRT), practice pattern trends, and overall survival (OS) were analyzed from the population-based data.

**Results:**

Over 9 years, 1,684 patients underwent surgical resection (74.9%) or SBRT (25.1%) as a localized treatment. From 2008 to 2016, the treatment modality changed: the percentage of patients who underwent surgical resection decreased from 90.6 to 71.4%, and those who underwent SBRT increased from 9.4 to 28.6%. The percentage of patients treated with SBRT increased over time (*p* < 0.001). The median OS was 56.4 months in the surgery group and 35.5 months in the SBRT group. The SBRT group showed worse OS compared with the surgery group (Adjusted hazard ratio, 1.44; 95% confidence interval, 1.21–1.72; *p* < 0.001).

**Conclusion:**

Changes in local treatment patterns in elderly lung cancer patients were observed and SBRT increased its role in this population. Surgical resection or SBRT should be considered the treatment of choice in elderly patients with localized lung cancer. Further prospective studies are required to elucidate the benefits of surgery and SBRT.

## Background

Lung cancer is the leading cause of cancer-related deaths in South Korea, although the 5-year survival rate has increased from 11.3 to 25.1% [1]. Lung cancer develops two or three times more frequently in individuals 70 years and older than in younger individuals, and it is the leading cause of cancer-related deaths in those aged 80 years and older [2]. Because the incidence of cancer is expected to increase among the elderly [3], it will be important to improve patient survival, especially in this population.

Surgical resection for early-stage lung cancer is the best treatment to improve survival. However, elderly patients (> 80 years old) with comorbidities do not easily receive surgery due to concerns about surgery-related mortality and morbidity [4–6]. Additionally, patients at this age often abandon the opportunity to cure early-stage non-small-cell lung cancer (NSCLC) due to the number of comorbidities, which increase proportionally with age [7, 8]. Stereotactic body radiation therapy (SBRT) is an alternative treatment modality that destroys lesions by delivering a high dose of radiation to the target site. SBRT showed a high local control rate comparable to lobectomy in early-stage NSCLC patients who were not eligible for surgery, with tolerable toxicities in patients with underlying emphysema and vascular diseases [9, 10]. The excellent local control and lower complication rates of SBRT may lead to changes in practical treatment patterns in patients with early-stage lung cancer who are not suitable for surgery or refuse treatment due to concerns over surgical complications. Toxicity issues associated with the treatment of early-stage NSCLC in the elderly remain, although previous studies have suggested that SBRT is safe and tolerable in elderly populations [11]. A recent study reported that the practice pattern and treatment outcomes have changed in elderly stage I lung cancer patients since the introduction of SBRT in the United States [12]. However, there was no report of annual survival outcomes for surgery and SBRT in a limited cohort of 80 years and older patients with early-stage lung cancer using a population-based database from South Korea.

This study evaluated the trends in practice patterns and survival outcomes over 9 years in patients 80 years and older diagnosed with early-stage lung cancer and compared the overall survival (OS) rates of those who underwent surgery and SBRT. We used a national claims database provided by the Health Insurance Review and Assessment service (HIRA).

## Methods

### Data source

The HIRA database, a nationwide claims database, covers the claims of 100% of the South Korean population. The HIRA database includes patient demographics and the record of diagnosis (as determined by the International classification of Disease, Tenth Revision), interventions, and prescriptions. The HIRA service provided the data after patient de-identification, in accordance with the Act on the Protection of Personal Information maintained by public agencies. This study was conducted in accordance with the Declaration of Helsinki. The Institutional Review Board of Samsung Medical Center approved this retrospective cohort study (Approval no. 2018-02-023); informed consent from patients was waived by the Board.

### Patient selection

Patients 80 years and older with a new diagnosis of lung cancer between January 2008 and December 2016 were collected from the HIRA database for possible inclusion. Lung cancer outcomes were identified on the basis of insurance claims data selected as the major diagnosis code in in-patient records (C34). Patients 80 years and older who underwent lung cancer surgery (lobectomy, segmentectomy, or wedge resection) or SBRT were included. Patients who underwent both surgery and SBRT, and who had a history of chemotherapy before SBRT or surgery were excluded. Patients who received any type of chemotherapy within 90 days after surgery or SBRT were also excluded. It was possible to retrieve only the HIRA data of patients who claimed treatments for lung cancer. We could not identify patients who had lung cancer with no treatments. Early-stage lung cancer was defined as cases of surgery or SBRT only without neoadjuvant/adjuvant treatment. The index date was defined as the first recorded date of surgery or SBRT. Cancer-directed treatment was regarded when the treatment occurred within 3 months after the diagnosis of lung cancer.

### Statistical analysis

Descriptive statistical analyses were performed on all patients. Continuous and categorical variables are presented as the mean ± standard deviation (SD) and numbers (%). Baseline characteristics among the two groups (operation vs. SBRT) were compared using the *t*-test for continuous variables and Pearson’s χ^2^ test for categorical variables. Univariate and multivariate Cox proportional hazard models were used to reveal the association between clinical variables and survival outcomes. These results are reported as hazard ratios (HRs) with 95% confidence intervals (CIs). Survival curves were calculated using the Kaplan-Meier method and were compared using the log-rank test. A two-tailed *p*-value of < 0.05 was considered to indicate statistical significance. All statistical analyses were performed using R program version 3.4.0.

## Results

### Patient characteristics

In total, 1,684 patients diagnosed with early-stage lung cancer from 2008 to 2016 and who met the inclusion criteria were eligible for this study. Of those, 1,262 (74.9%) patients underwent surgical resection and 422 (25.1%) underwent SBRT. The clinical characteristics are summarized in Table 1. The mean age at lung cancer diagnosis was 82.0 ± 2.2 years in the surgery group and 83.2 ± 3.1 years in the SBRT group. Males accounted for 71.6% of the subjects in the surgery group and 71.8% in the SBRT group. In total, 91.3% of patients in the surgery group and 91.9% of patients in the SBRT group did not receive any type of chemotherapy during the entire follow-up period. Only 8.7% of individuals in the surgery group and 8.1% of individuals in the SBRT group received chemotherapy after 3 months of local treatment. There was no significant difference in clinical treatment failure represented as additional chemotherapy between the two groups (*p* = 0.750). The median length of follow-up was 20.4 months (interquartile range [IQR], 7.4–40.3 months) for the surgery group and 16.8 months (IQR, 6.9–30.0 months) for the SBRT group.

**Table 1:** Demographics of very elderly (≥80 years old) lung cancer patients

Characteristic	Treatment, No. (%)		
	Total (*N* = 1,684)	Surgery (*N* = 1,262)	SBRT (*N* = 422)
Mean age, years	82.3 ± 2.5	82.0 ± 2.2	83.2 ± 3.1
Sex			
Male	1,207 (71.7)	904 (71.6)	303 (71.8)
Female	477 (28.3)	358 (28.4)	119 (28.2)
Chemotherapy^a^			
No	1,540 (91.4)	1,152 (91.3)	388 (91.9)
Yes	144 (8.6)	110 (8.7)	34 (8.1)
Year of treatment			
2008	64	58 (90.6)	6 (9.4)
2009	83	72 (86.7)	11 (13.3)
2010	105	89 (84.8)	16 (15.2)
2011	111	90 (81.1)	21 (18.9)
2012	186	139 (74.7)	47 (25.3)
2013	219	154 (70.3)	65 (29.7)
2014	255	188 (73.7)	67 (26.3)
2015	297	212 (71.4)	85 (28.6)
2016	364	260 (71.4)	104 (28.6)
Follow-up, months			
Median	19.4	20.4	16.8
IQR	7.2–36.5	7.4–40.3	6.8–30.0
Median OS, months			
Median	49.8	56.4	35.5
95% CI	44.7–56.3	49.1–66.5	28.9–41.7
30-day mortality			
No. (%)	36 (2.2)	30 (2.4)	6 (1.5)
95% CI	1.5–2.9	1.6–3.3	0.3–2.6

^a^Defined as patients who received any type of chemotherapy after 3 months of local treatment

All values are presented as the number (%). *N* number, *OS* overall survival, *CI* confidence interval, *SBRT* stereotactic body radiation therapy

### Temporal changes in local treatment patterns

**Fig. 1:**
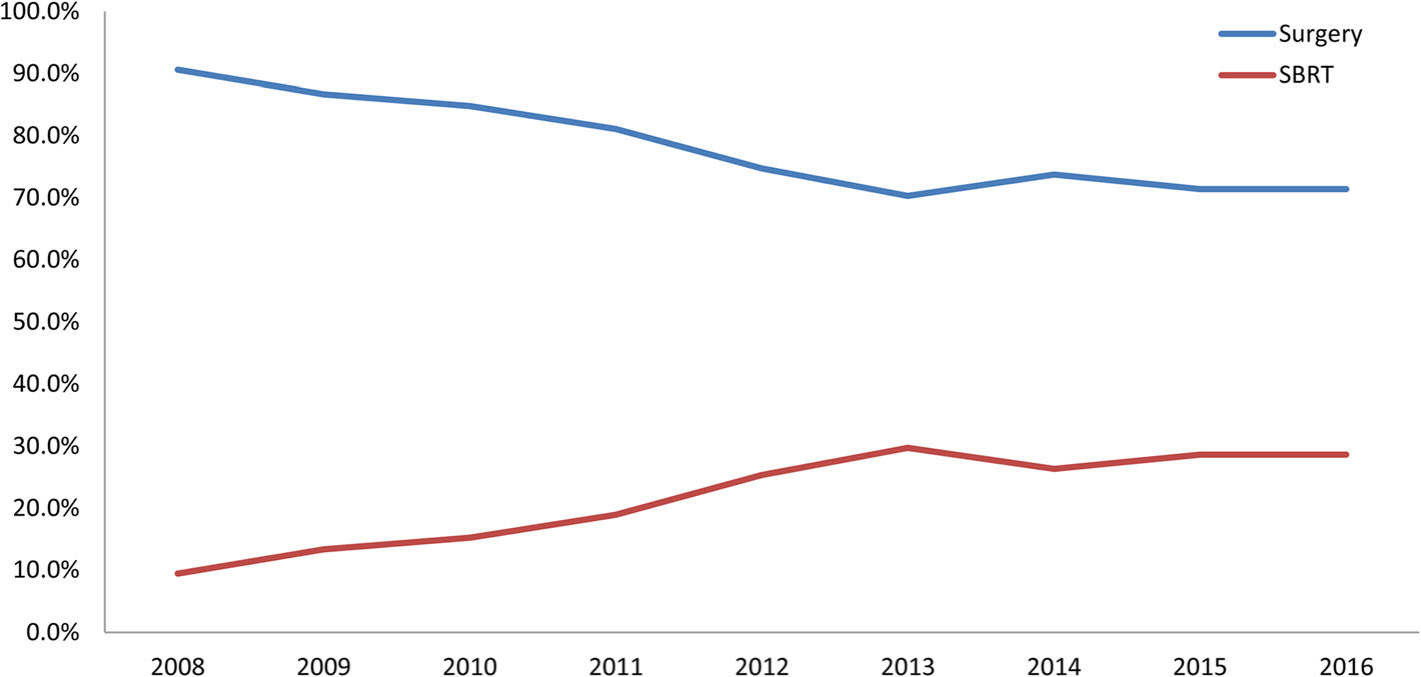
Practice changes in local treatment patterns in very elderly (≥80 years old) patients diagnosed with early-stage lung cancer. The proportion of patients who underwent stereotactic body radiation therapy (SBRT) increased gradually as a local treatment modality in the old age group over the year (*p* < 0.001)

During the study period, the use of local treatment increased among the elderly patients with early-stage lung cancer from 64 patients in 2008 to 364 patients in 2016. The trends in treatment practice change between 2008 and 2016 are shown in Fig. 1. The proportion of patients who underwent surgery declined continuously from 90.6 to 71.4%; however, the use of SBRT increased from 9.4% in 2008 to 28.6% in 2016 (*p* < 0.001).

### Survival outcomes and predictive factors for elderly lung cancer

**Fig. 2:**
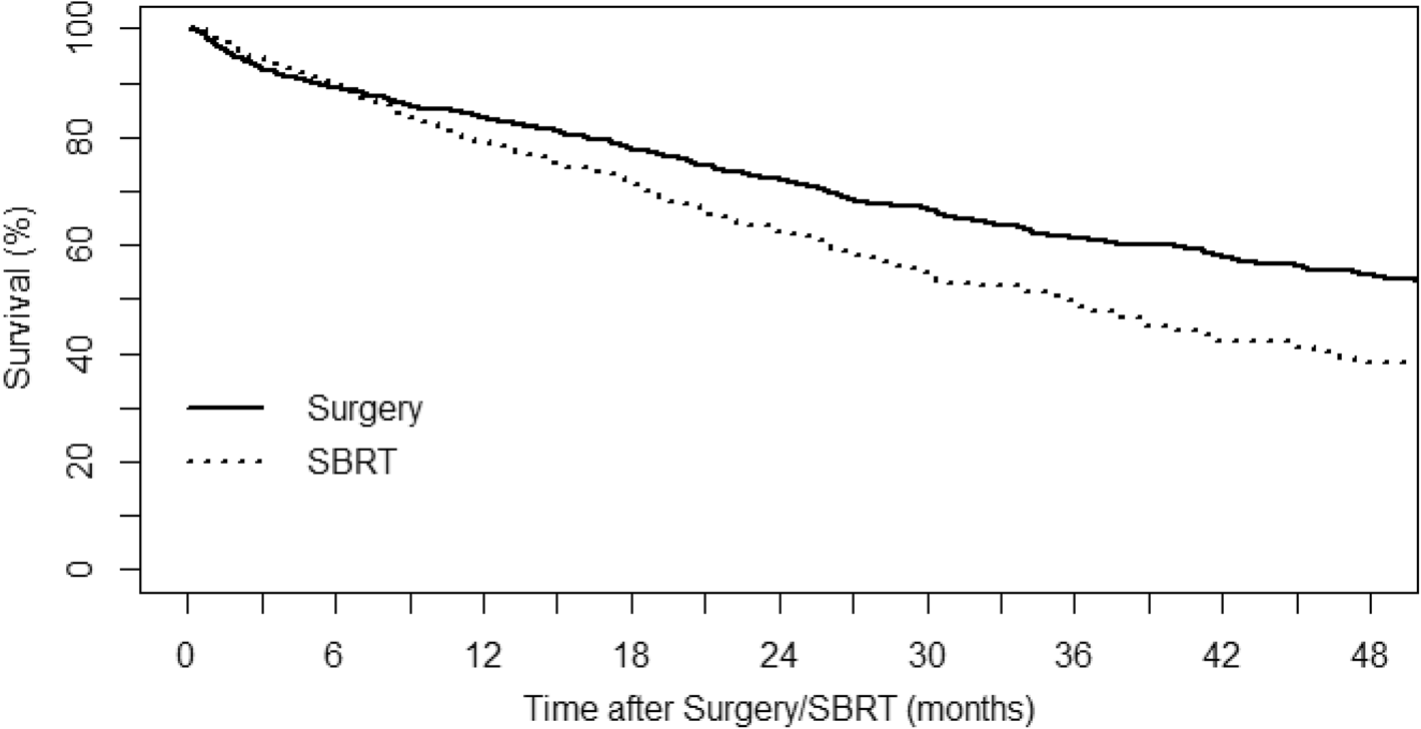
Kaplan-Meier curves for overall survival according to treatment modality in very elderly (≥80 years) patients with early-stage lung cancer (*p* < 0.001, log-rank test). SBRT, stereotactic body radiation therapy

Compared with the SBRT group, OS was higher in the surgery group (2-year OS, 72.2% for the surgery group vs. 62.6% for the SBRT group, *p* = 0.005). The median survival time was 56.4 months in the surgery group and 35.5 months in the SBRT group. Post-treatment mortality at 30 days was 2.4% for the surgery group and 1.5% for the SBRT group. At 6 months after local treatment, the rate of mortality was 11.0% in the surgery group and 10.7% in the SBRT group. The comparison of OS between the surgery and SBRT groups from 2008 to 2016 is presented in Fig. 2. To determine the predictive factors for survival in elderly lung cancer patients, a Cox’s regression analysis was performed using clinical factors. Clinical variables for the univariate and multivariate analyses are listed in Table 2. According to the univariate analysis, male sex, chemotherapy after 3 months of local treatment, and SBRT were associated with decreased OS. However, patient age in the elderly population (> 80 years) was not a predictive factor. Sex, chemotherapy after 3 months of local treatment, and SBRT remained significantly associated with OS after the multivariate analysis. In terms of treatment modality, the adjusted HR for SBRT was 1.44 (95% CI, 1.21–1.72) with a reference to surgery.

**Table 2:** Hazard model of survival outcomes in patients with early-stage lung cancer

Variable	Univariate analysis			Multivariate analysis		
	HR	95% CI	*p*-value	HR	95% CI	p-value
Age, years						
80–84	Reference					
85–90	1.12	0.90–1.40	0.302			
> 90	1.11	0.67–1.86	0.686			
Sex						
Male	Reference			Reference		
Female	0.42	0.34–0.52	< 0.001	0.41	0.33–0.50	< 0.001
Treatment year						
2008	1.36	0.83-2.22	0.225			
2009	1.49	0.94–2.39	0.091			
2010	1.75	1.23–2.73	0.013			
2011	1.15	0.73–1.82	0.548			
2012	1.15	0.74–1.77	0.532			
2013	1.44	0.95–2.19	0.089			
2014	1.07	0.69–1.64	0.769			
2015	0.97	0.62–1.51	0.882			
2016	Reference					
Chemotherapy						
No	Reference			Reference		
Yes	1.92	1.47–2.26	< 0.001	1.95	1.54–2.39	< 0.001
Treatment modality						
Surgery	Reference			Reference		
SBRT	1.44	1.21–1.72	< 0.001	1.44	1.21–1.72	< 0.001

*HR* hazard ratio, *CI* confidence interval, *SBRT* stereotactic body radiation therapy

## Discussion

In this population-based study, we uncovered temporal practice changes in patients 80 years and older diagnosed with early-stage lung cancer between 2008 and 2016 in real practice in South Korea. Although most patients received surgical resection (74.9%), there were significant changes in treatment trends at the time of the study period. Compared to 2008, the rate of surgical resection decreased gradually from 90.6 to 71.4%. However, the rate of SBRT increased over time from 9.4% in 2008 to 28.6% in 2016. The standard treatment for early-stage lung cancer is surgical resection with lobectomy [13]. However, in cases of poor surgical candidates with old age and comorbidities, it is not easy to perform surgical resection because of the high risk of complications. Since the introduction of SBRT for non-operable lung cancer due to comorbidities such as severe emphysema or heart disease [14], it has been adopted in clinical practice as an alternative to surgery accompanied by expected high risks of complications in patients with early-stage lung cancer. Practice pattern changes in elderly stage I NSCLC patients in which SBRT replaced surgery based on older age is now recognized in the United States [12]. Our data also suggest that SBRT increased its proportion substantially in elderly early-stage lung cancer patients in South Korea.

In our study, the median OS and 5-year survival rate in patients 80 years and older was 49.8 months and 44.8% with local treatment for early-stage lung cancer, respectively. A large population-based study analyzed 101,844 cases of lung cancer registered in the California Cancer Center for 4 years; stage 1 lung cancer was diagnosed in 19,702 patients. Of these, 7.3% of patients did not receive surgery or any type of radiation or chemotherapy. In this untreated group, the median OS and 5-year survival rate was 9 months and 6%, respectively [15]. In particular, lung cancer in the elderly has shown increased mortality, regardless of stage [16]. Therefore, screening for lung cancer with low-dose computed tomography [17] may be considered for those older than 80 years to reduce mortality in at-risk patients, although the evidence is not sufficient. However, there are concerns that old age makes treatment decisions difficult due to increased comorbidities and decreased physiologic performance. In advanced-stage NSCLC, a substantial number of patients were not treated and older patients were more likely to not receive any treatment [18]. Patients 80 years and older are less likely to be subjected to local treatment compared to younger patients; this results in a poor survival rate in older patients with lung cancer. Although we were unable to compare the survival rate between patients who received treatment and those who did not, definitive local treatment with surgery or SBRT should be performed in patients 80 years and older to increase the survival rate.

Our study shows that surgical resection had higher survival outcomes compared with SBRT after adjusting for variables using these population-based data. However, this result does not represent the head-to-head comparison of OS between the two treatment modalities. SBRT is generally adopted for patients who are not surgically suitable. In any nationwide claims study, selection bias is inevitable because it is impossible to obtain information regarding stage I or II cases, Eastern Cooperative Oncology Group (ECOG) performance, and factors related to surgical risk. Comorbidities increase with age and are the major reason for elderly patients with increased comorbidities to be precluded from surgical resection in cases of early-stage lung cancer [19]. Published surgical mortality and morbidity rates in patients 80 years and older range from 2.0 to 3.6% and 8.4 to 46%, respectively [4, 5, 20]. Although the Radiation Therapy Oncology Group confirmed SBRT as an alternative treatment modality for inoperable early-stage lung cancer [9], there are concerns about the safety and efficacy of SBRT in patients 80 years and older. A multicenter study reported that grade 2 radiation pneumonitis (RP) developed in 34.5% of patients and non-RP related toxicity was reported in 12.1%; however, no grade 4 or 5 toxicity was reported in the group who underwent SBRT for early-stage lung cancer (median age, 84.9 years) [21]. Another study regarding the safety of definitive SBRT in patients more than 80 years old suggested its high efficacy and tolerability [11]. Of interest, a prior study that investigated the effectiveness of SBRT at a single center reported that the OS rates for patients over 75 years were 86% at 1 year, 57.5% at 3 years, and 39.5% at 5 years [22], which is comparable to the survival rates of 79.1% at 1 year, 61.3% at 3 years, and 32.4% at 5 years in our patients who underwent SBRT.

Local relapse or distant metastasis is an important issue in early-stage lung cancer with definitive local treatment. The overall recurrence (local/regional/distant) reported in previous studies for elderly patients with SBRT was 30–40% [21, 23]. Therefore, the 8–9% rate of systemic chemotherapy after 3 months of local treatment in our study may be relatively low, in which surgery or SBRT revealed no significant difference in systemic chemotherapy after 3 months of definitive local treatment. This relatively low chemotherapy rate may be explained by old age-related comorbidities or poor ECOG performance. Although it should not be interpreted as a parameter of recurrence, we may indirectly estimate the rate of systemic chemotherapy as treatment failure after local control and suggest similar rates of treatment failure between the two modalities.

Our study has several limitations. A major limitation of nationwide claims data is the lack of clinical information. We could not obtain information related with performance status, underlying lung function with chronic obstructive pulmonary disease, interstitial lung disease, and accompanied comorbidities. Second, lung cancer stage (I or II) was not clearly divided in this study. The HIRA database contains only information related to claims covered by national insurance. Therefore, it was impossible to stratify the lung cancer staging according to the clinical information. Based on the claims database, we could retrieve early-stage lung cancer data only indirectly using patients who underwent surgical resection only or stereotactic body radiation therapy (SBRT) with no neoadjuvant treatment or adjuvant chemotherapy within 3 months after local treatment. Finally, local recurrence and distant metastasis could not be measured. Only data regarding chemotherapy 3 months later could be obtained; therefore, the rate of treatment failure may be underestimated.

## Conclusions

The rate of SBRT increased gradually in patients 80 years and older with early-stage lung cancer, in contrast with the decrease in surgical resection. Surgical resection or SBRT is a reasonable treatment choice for elderly patients with early-stage lung cancer and should be adopted in light of multifactorial decision making.

## Abbreviations

ECOG: Eastern Cooperative Oncology Group
HIRA: Health insurance review and assessment service
HRs: Hazard ratios
NSCLC: Non-small-cell lung cancer
OS: Overall survival
SBRT: Stereotactic body radiation therapy
